# A study of the relationship between serum asprosin levels and MAFLD in a population undergoing physical examination

**DOI:** 10.1038/s41598-024-62124-w

**Published:** 2024-05-15

**Authors:** Dan Lv, Zepu Wang, Cuiqiao Meng, Yan Li, Shuai Ji

**Affiliations:** 1 Physical Examination Center, Hebei General Hospital, Shijiazhuang City, Hebei Province China; 2 Department of Hepatobiliary Surgery, Hebei General Hospital, Shijiazhuang City, Hebei Province China

**Keywords:** Asprosin, Controlled attenuation parameter, Metabolic-associated fatty liver disease, Transient elastography, Metabolic, Endocrinology, Medical research

## Abstract

Asprosin, an adipokine, was recently discovered in 2016. Here, the correlation between asprosin and metabolic-associated fatty liver disease (MAFLD) was examined by quantitatively assessing hepatic steatosis using transient elastography and controlled attenuation parameter (CAP). According to body mass index (BMI), 1276 adult participants were enrolled and categorized into three groups: normal, overweight, and obese. The study collected and evaluated serum asprosin levels, general biochemical indices, liver stiffness measure, and CAP via statistical analysis. In both overweight and obese groups, serum asprosin and CAP were greater than in the normal group (*p* < 0.01). Each group showed a positive correlation of CAP with asprosin (*p* < 0.01). The normal group demonstrated a significant and independent positive relationship of CAP with BMI, low-density lipoprotein cholesterol (LDL-C), asprosin, waist circumference (WC), and triglycerides (TG; *p* < 0.05). CAP showed an independent positive association (*p* < 0.05) with BMI, WC, asprosin, fasting blood glucose (FBG), and TG in the overweight group, and with high-density lipoprotein cholesterol (HDL-C) showed an independent negative link (*p* < 0.01). CAP showed an independent positive relationship (*p* < 0.05) with BMI, WC, asprosin, TG, LDL-C, FBG, glycated hemoglobin A1c (HbA1c), and alanine transferase in the obese group. CAP also showed an independent positive link (*p* < 0.01) with BMI, WC, asprosin, TG, LDL-C, and FBG in all participants while independently and negatively correlated (*p* < 0.01) with HDL-C. Since asprosin and MAFLD are closely related and asprosin is an independent CAP effector, it may offer a novel treatment option for metabolic diseases and MAFLD.

## Introduction

Non-alcoholic fatty liver disease (NAFLD) is also referred to as metabolic-associated fatty liver disease (MAFLD), including hepatic features and probable metabolic abnormalities. Due to the growing number of individuals with obesity and its related metabolic syndrome, MAFLD has emerged as an important contributor to chronic liver disease on a global scale. The disease's prevalence has been estimated to range from 10 to 24%, varying depending on the country. Among obese populations, the prevalence of MAFLD is estimated to be between 57.5 and 74%^1^. MAFLD is a medical condition defined by the abnormal accumulation of fat within liver cells. The main reasons contributing to its development are hyperinsulinemia, resistance to insulin, increased lipolysis in peripheral tissues, and an imbalance of the redox system causing oxidative stress^2–4^.

Currently, liver biopsy continues to be the most reliable method for determining the histologic staging and grading of MAFLD. However, liver biopsy is a process that is considered invasive and has a certain degree of risk for potential complications. Given the remarkably high occurrence of MAFLD, it is challenging to conduct a comprehensive liver biopsy on such a large number of individuals. Consequently, other non-invasive approaches are necessary to evaluate the extent of MAFLD. Transient elastography (TE) or Fibroscan (Echosens, Paris, France) quantifies hepatic steatosis by measuring the controlled attenuation parameter (CAP)^5–8^. In an initial study examining the application of CAP for assessing hepatic steatosis in individuals with chronic liver disease, it was found that this technique had a high level of accuracy in diagnosing the condition. It was reported that area under the receiver operating characteristic curve (AUROCs) of 0.91, 0.95, and 0.89 for detecting hepatic steatosis were > 10%, > 33%, and > 66%, respectively^9^. Subsequently, the accuracy and consistency of CAP in evaluating hepatic steatosis have been verified^10,11^.

Asprosin is a gluconeogenic and pro-phagocytic adipokine newly identified in 2016 by Romero et al. It is primarily released into the bloodstream by white adipose tissue and affects the liver^12^. Recent research has indicated that asprosin can cause inflammation and stress in the endoplasmic reticulum. Additionally, it has been observed that levels of asprosin in the bloodstream are higher in specific metabolic disorders such as type 2 diabetes mellitus (T2DM), obesity, polycystic ovary syndrome, gestational diabetes mellitus, and cardiometabolic diseases^13^. The association of MAFLD with insulin resistance, metabolic syndrome, and endoplasmic reticulum stress is widely recognized. However, there is a lack of research on the correlation between asprosin and MAFLD. There appears to be a close relationship between asprosin and MAFLD. Understanding this relationship can more convenient aid in the early diagnosis of MAFLD through asprosin, potentially preventing progression to liver fibrosis, cirrhosis, and end-stage liver disease. Asprosin may be a promising target for treating MAFLD and related metabolic diseases, and could lead to the development of new treatment plans.

CAP was used as a versatile diagnostic tool in this cross-sectional study to assess the prevalence and severity of hepatic steatosis. Furthermore, the connection between asprosin and MAFLD was investigated as well.

## Materials and methods

### Study population

Hebei General Hospital's ethical committee gave its approval, and the research followed all the rules provided in the Declaration of Helsinki. Informed consent was obtained from all the participants. The formula n = 100 + 50(i) is employed to calculate the sample size of multiple linear regression, where i refers to the number of independent variables in the final model^14,15^. This research recruited 1276 adults who underwent health checkups at the Physical Examination Center of Hebei General Hospital from January 2022 to January 2023. According to the guidelines of the Working Groups on Obesity in China^16^, all research participants were classified into normal group (BMI < 24 kg/m^2^), overweight group (24 kg/m^2^ ≤ BMI < 28 kg/m^2^), and obese group (BMI ≥ 28 kg/m^2^). Out of the total participants, 313 were in the normal group, 600 were in the overweight group, and 363 were in the obese group. Exclusion of individuals was based on smoking and alcohol-drinking status and the presence of other medical conditions. The following are the exclusion criteria: (1) chronic viral hepatitis, (2) chronic kidney disease and blood systemic diseases, (3) acute cardiovascular disease, (4) using or having used drugs that affect liver metabolism within the last 6 months, (5) drink more than 140 g/week for men and 70 g/week for women, and (6) pregnancy.

### Data collection

The physician, having received uniform training, gathered data on age and sex from all participants in the trial. Participants' weight, waist circumference (WC), and height were measured per a predetermined procedure. The body mass index (BMI) was calculated per the standard formula.

### Blood sample collection and measurement

The research participants were instructed to abstain from eating for 12 h before blood collection. Subsequently, 10 mL of blood was acquired from the participant's elbow vein in the morning. Afterwards, the samples were promptly kept at 4 °C, and the plasma was isolated using centrifugation at a speed of 4000 rpm. The plasma samples obtained were kept at − 80 °C. Aspartate transferase (AST), triglycerides (TG), fasting blood sugar (FBS), low-density lipoprotein cholesterol (LDL-C), total cholesterol (TC), alanine transferase (ALT), and high-density lipoprotein cholesterol (HDL-C) were quantified using an automated biochemistry analyzer. Furthermore, glycated hemoglobin (HbA1c) was assessed via high-performance liquid chromatography (HPLC) with an automated system. Platelet counts (PLT) were determined using an automated hematology analyzer.

### Determination of asprosin

Serum Asprosin levels were evaluated via ELISA, following the manufacturer's instructions. The kit was purchased from ELAAB Science Inc. (E15190h), Wuhan, China. The test samples exhibited coefficients of variation between batches and between intra- and inter-batch, ranging from less than 10% to less than 12%.

### Measurement of liver stiffness (LSM) and CAP

Technicians utilized Fibroscan (Echosens, Paris, France) to conduct more than 500 tests and quantified LSM and CAP. To expose the intercostal space on the liver's right lobe, the participant assumed a supine position while raising their right arm. The probe was placed perpendicular to the skin to measure in the intercostal gap. Initially, measurements were done using a standard 3.5 MHz probe, sometimes known as a M probe. The XL probe measures were conducted as a contingency when M probe measurements were ineffective, particularly in cases involving obese individuals. Measurements were deemed valid if the ratio of the quartile spacing to the median was below 30%. The participants endured 10 successful measures that were verified as accurate, and the final result was determined by computing the median of those ten valid measurements. The results were expressed in decibels per meter (dB/m) for CAP and kilopascals (kPa) for LSM.

### Statistical analysis

SPSS version 21.0 was utilized for all analyses. Normally distributed data were shown as mean ± standard deviation (SD) and measured via the analysis of variance (ANOVA) for multi-group comparisons. The rank-sum test was utilized to assess non-normally distributed data, which were denoted as M (P25, P75), to compare them between groups. Numbers or percentages were utilized to indicate categorical variables, and the chi-square test was employed for group comparisons. The associations between Asprosin and CAP were investigated using Pearson or Spearman correlation analysis. Multiple linear regression was used to do multifactorial analysis. Statistical data were deemed significant if *p* < 0.05.

## Results

### Clinical features of all participants

This study included 1276 individuals (936 males and 340 females). The normal group comprised 313 participants with a BMI < 24 kg/m^2^. The overweight group had 600 participants with a BMI between 24 and 28 kg/m^2^. The obese group had 363 participants with a BMI ≥ 28 kg/m^2^. Details of age, sex, WC, and liver function parameters of the participants in the three groups are shown in Table 1.

**Table 1:** Clinical characteristics of all participants.

	Normal (n = 313)	Overweight (n = 600)	Obesity (n = 363)	*P*
Age (years)	49.17 ± 11.35	50.38 ± 10.78	49.12 ± 10.40	0.125
Sex (male/female)	161/152	466/134	309/54	< 0.001**
WC (cm)	81.11 ± 6.65	91.48 ± 6.91	102.39 ± 7.25	< 0.001**
CAP (dB/m)	229.51 ± 42.22	267.54 ± 44.80	305.74 ± 42.55	< 0.001**
LSM (kPa)	4.29 ± 0.85	4.49 ± 0.92	5.07 ± 0.94	< 0.001**
Asprosin (ng/mL)	17.44 ± 8.01	24.54 ± 9.02	39.01 ± 8.97	< 0.001**
TG (mmol/L)	1.19 (0.85, 1.68)	1.52 (1.09, 2.23)	1.88 (1.42, 2.77)	< 0.001**
TC (mmol/L)	5.12 ± 1.11	5.27 ± 1.02	5.42 ± 1.03	0.001**
LDL-C (mmol/L)	3.22 ± 0.72	3.33 ± 072	3.49 ± 0.73	< 0.001**
HDL-C (mmol/L)	1.48 ± 0.28	1.25 ± 0.25	1.17 ± 0.27	< 0.001**
FBG (mmol/L)	5.27 (4.99, 5.70)	5.63 (5.16, 6.20)	5.70 (5.28, 6.48)	< 0.001**
HbA1c (%)	5.60 (5.40, 5.90)	5.80 (5.50, 6.10)	5.90 (5.60, 6.30)	< 0.001**
PLT (× 10^9^/L)	251.37 ± 52.37	248.58 ± 51.43	245.90 ± 51.36	0.388
ALT (IU/L)	17.00 (12.70, 23.90)	22.50 (16.13, 31.58)	24.70 (19.30, 36.40)	< 0.001**
AST (IU/L)	20.40 (17.60, 24.55)	21.50 (18.60, 26.30)	22.50 (18.80, 26.60)	< 0.001**

*P < 0.05, **P < 0.01.

### Comparison of CAP and serum asprosin levels in different groups

**Figure 1:**
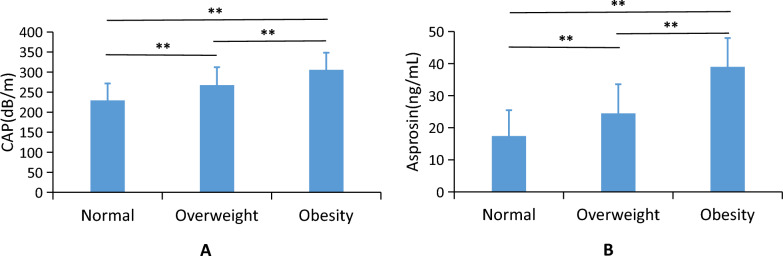
Comparison of CAP and serum asprosin levels in different groups.

CAP values were greater in both the overweight and obese in comparison to the normal group (*p* < 0.01). Additionally, the obese group showed a substantially higher CAP level in comparison to the overweight group (*p* < 0.01; Table 1, Fig. 1A). Furthermore, the asprosin levels in serum were significantly greater in both the overweight and obese groups in comparison to the normal group (*p* < 0.01). Moreover, the obese group had higher serum concentrations of asprosin than that of the overweight group (*p* < 0.01; Table 1, Fig. 1B).

### Correlation analysis of asprosin with different indicators

The levels of asprosin in all participants showed significant positive correlations with BMI, WC, LSM, TG, TC, LDL-C, FBG, HbA1c, ALT, and AST (*r* = 0.680, *r* = 0.602, *r* = 0.274, *r* = 0.429, *r* = 0.249, *r* = 0.276, *r* = 0.352, *r* = 0.362, *r* = 0.263, and *r* = 0.151, respectively, *p* < 0.01) and correlated negatively with HDL-C (*r* = − 0.357, *p* < 0.01; Table 2).

**Table 2:** Correlation analysis of asprosin with different indicators.

	Normal (n = 313)		Overweight (n = 600)		Obesity (n = 363)		Overall (n = 1276)	
	*r*	*P*	*r*	*P*	*r*	*P*	*r*	*P*
Age	0.034	0.547	0.025	0.541	− 0.040	0.447	− 0.005	0.851
BMI	0.188	0.001**	0.203	< 0.001**	0.349	< 0.001**	0.680	< 0.001**
WC	0.194	0.001**	0.228	< 0.001**	0.217	< 0.001**	0.602	< 0.001**
CAP	0.312	< 0.001**	0.331	< 0.001**	0.423	< 0.001**	0.574	< 0.001**
LSM	0.017	0.769	0.040	0.332	0.200	< 0.001**	0.274	< 0.001**
TG	0.365	< 0.001**	0.312	< 0.001**	0.352	< 0.001**	0.429	< 0.001**
TC	0.250	< 0.001**	0.242	< 0.001**	0.258	< 0.001**	0.249	< 0.001**
LDL-C	0.224	< 0.001**	0.251	< 0.001**	0.271	< 0.001**	0.276	< 0.001**
HDL-C	− 0.110	0.053	− 0.195	< 0.001**	− 0.233	< 0.001**	− 0.357	< 0.001**
FBG	0.299	< 0.001**	0.303	< 0.001**	0.369	< 0.001**	0.352	< 0.001**
HbA1c	0.342	< 0.001**	0.285	< 0.001**	0.446	< 0.001**	0.362	< 0.001**
PLT	− 0.041	0.472	0.036	0.382	0.051	0.331	− 0.009	0.822
ALT	0.053	0.353	0.098	0.017*	0.252	< 0.001**	0.263	< 0.001**
AST	0.022	0.699	0.085	0.037*	0.226	< 0.001**	0.151	< 0.001**

*P < 0.05, **P < 0.01.

In the normal group, asprosin exhibited a positive significant correlation with BMI, WC, TG, TC, LDL-C, FBG, and HbA1c (*r* = 0.188, *r* = 0.194, *r* = 0.365, *r* = 0.250, *r* = 0.224, *r* = 0.299, and *r* = 0.342, respectively, *p* < 0.01; Table 2).

In the overweight group, asprosin demonstrated a positive significant relationship with BMI, WC, TG, TC, LDL-C, FBG, HbA1c, ALT, and AST (*r* = 0.203, *r* = 0.228, *r* = 0.312, *r* = 0.242, *r* = 0.251, *r* = 0.303, *r* = 0.285, *r* = 0.098, and *r* = 0.085, respectively, *p* < 0.05). Moreover, a negative association was observed with HDL-C (*r* = − 0.195, *p* < 0.01; Table 2).

In the obese group, asprosin had a positive link with BMI, WC, LSM, TG, TC, LDL-C, FBG, HbA1c, ALT, and AST (*r* = 0.349, *r* = 0.217, *r* = 0.200, *r* = 0.352, *r* = 0.258, *r* = 0.271, *r* = 0.369, *r* = 0.446, *r* = 0.252, and *r* = 0.226, *p* < 0.01) and correlated negatively with HDL-C (*r* = − 0.233, *p* < 0.01; Table 2).

### Correlation analysis of CAP with different indicators

CAP negatively correlated with HDL-C (*r* = − 0.395, *p* < 0.01) and positively correlated with BMI, WC, LSM, TG, TC, LDL-C, FBG, HbA1c, and AST(*r* = 0.627, *r* = 0.662, *r* = 0.265, *r* = 0.568, *r* = 0.243,* r* = 0.281, *r* = 0.451, *r* = 0.365, and *r* = 0.235, *p* < 0.01) in all study participants as shown in Table 3.

**Table 3:** Correlation analysis of CAP with different indicators.

	Normal (n = 313)		Overweight (n = 600)		Obesity (n = 363)		Overall (n = 1276)	
	*r*	*P*	*r*	*P*	*r*	*P*	*r*	*P*
Age	0.105	0.064	0.005	0.910	− 0.075	0.151	0.004	0.875
BMI	0.459	< 0.001**	0.397	< 0.001**	0.456	< 0.001**	0.627	< 0.001**
WC	0.538	< 0.001**	0.447	< 0.001**	0.440	< 0.001**	0.662	< 0.001**
Asprosin	0.312	< 0.001**	0.331	< 0.001**	0.423	< 0.001**	0.574	< 0.001**
LSM	0.085	0.133	0.088	0.030*	0.227	< 0.001**	0.265	< 0.001**
TG	0.495	< 0.001**	0.528	< 0.001**	0.481	< 0.001**	0.568	< 0.001**
TC	0.282	< 0.001**	0.208	< 0.001**	0.197	< 0.001**	0.243	< 0.001**
LDL-C	0.360	< 0.001**	0.206	< 0.001**	0.222	< 0.001**	0.281	< 0.001**
HDL-C	− 0.223	< 0.001**	− 0.266	< 0.001**	− 0.243	< 0.001**	− 0.395	< 0.001**
FBG	0.312	< 0.001**	0.481	< 0.001**	0.511	< 0.001**	0.481	< 0.001**
HbA1c	0.298	< 0.001**	0.414	< 0.001**	0.532	< 0.001**	0.451	< 0.001**
PLT	0.051	0.371	0.041	0.311	− 0.043	0.418	− 0.004	0.900
ALT	0.268	< 0.001**	0.234	< 0.001**	0.307	< 0.001**	0.365	< 0.001**
AST	0.131	0.021*	0.178	< 0.001**	0.286	< 0.001**	0.235	< 0.001**

*P < 0.05, **P < 0.01.

Moreover, Table 3 also showed that in the normal group, CAP correlated negatively with HDL-C (*r* = − 0.223, *p* < 0.01) and a positive relationship with BMI, WC, TG, TC, LDL-C, FBG, HbA1c, ALT, and AST (*r* = 0.459, *r* = 0.538, *r* = 0.495, *r* = 0.282, *r* = 0.360, *r* = 0.312, *r* = 0.298, *r* = 0.268, and *r* = 0.131, respectively, *p* < 0.05).

CAP showed a positive correlation with BMI, WC, LSM, TG, TC, LDL-C, FBG, HbA1c, ALT, and AST (*r* = 0.397, *r* = 0.447, *r* = 0.088, *r* = 0.208, *r* = 0.206, *r* = 0.481, *r* = 0.234, and *r* = 0.178, respectively, *p* < 0.05) in the overweight group and correlated negatively with HDL-C (*r* = − 0.266, *p* < 0.01; Table 3).

In Table 3 the obese group reveals a positive link of CAP with BMI, WC, LSM, TG, TC, LDL-C, FBG, HbA1c, ALT, and AST (*r* = 0.456, *r* = 0.440, *r* = 0.227, *r* = 0.481, *r* = 0.197, *r* = 0.222, *r* = 0.511, *r* = 0.532, *r* = 0.307, and *r* = 0.286, respectively,* p* < 0.01). Conversely, there was a significant negative relationship (*r* = − 0.243, *p* < 0.01) of CAP with HDL-C.

### Correlation analysis between CAP and asprosin

**Figure 2:**
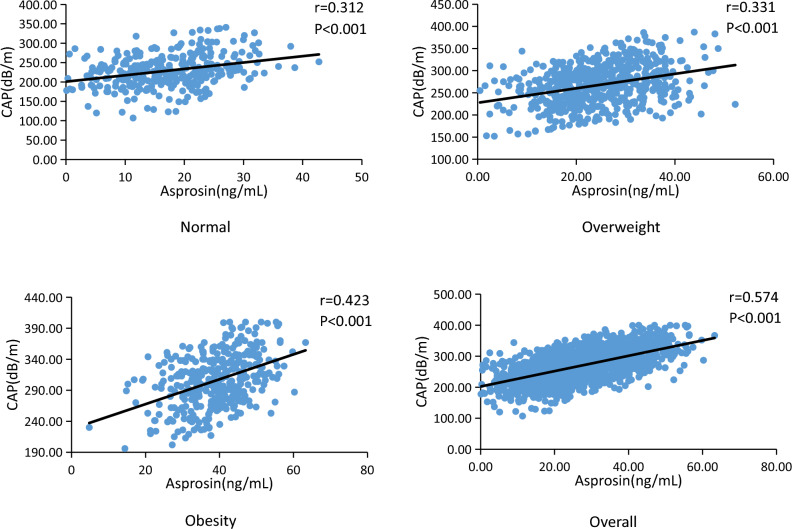
Correlation analysis between CAP and asprosin.

As shown by the correlation analysis, CAP and asprosin showed a significant positive correlation regardless of the group (*p* < 0.01). The value of *r* is 0.312 for the normal group (Fig. 2A), while it is 0.331 for the overweight group (Fig. 2B), 0.423 for the obese group (Fig. 2C), and 0.574 for all participants combined (Fig. 2D).

### Multivariate linear regression analysis

To investigate the independent association of CAP with each study parameter, a multivariate analysis was performed. The findings revealed that CAP was positively and independently linked to BMI, TG, asprosin, WC, and LDL-C in the normal group (*p* < 0.05; Table 4). Moreover, in the overweight group, CAP had an independent and positive relationship with BMI, WC, asprosin, TG, and FBG (*p* < 0.05). Conversely, CAP revealed an independent and negative association with HDL-C in the overweight group (*p* < 0.01; Table 4). The CAP was positively and independently linked with WC, TG, asprosin, BMI, FBG, LDL-C, HbA1c, and ALT (*p* < 0.05; Table 4) in the obese group. For all study participants, it was revealed that CAP had an independent and positive correlation with TG, asprosin, WC, LDL-C, BMI, and FBG (*p* < 0.01) and had an independent negative association with HDL-C (*p* < 0.01; Table 4).

**Table 4:** Multivariate linear regression analysis.

	Normal (n = 313)		Overweight (n = 600)		Obesity (n = 363)		Overall (n = 1276)	
	*t*	*P*	*t*	*P*	*t*	*P*	*t*	*P*
BMI	2.301	0.022*	5.247	< 0.001**	3.468	0.001**	5.177	< 0.001**
WC	5.268	< 0.001**	5.588	< 0.001**	3.588	< 0.001**	8.791	< 0.001**
Asprosin	2.169	0.031*	2.123	0.034*	2.253	0.025*	3.201	0.001**
LSM	1.091	0.276	− 0.110	0.913	0.599	0.550	0.407	0.684
TG	3.559	< 0.001**	2.403	0.017*	3.216	0.001**	5.648	< 0.001**
TC	− 0.077	0.938	0.673	0.501	− 0.143	0.886	− 0.304	0.761
LDL-C	2.763	0.006**	0.676	0.499	2.269	0.024*	4.434	< 0.001**
HDL-C	− 0.792	0.429	− 3.239	0.001**	− 0.441	0.659	− 3.488	0.001**
FBG	0.801	0.424	2.101	0.036*	2.092	0.037*	3.880	< 0.001**
HbA1c	− 0.423	0.672	1.920	0.055	1.988	0.048*	1.813	0.070
ALT	1.041	0.299	0.193	0.847	2.295	0.022*	1.689	0.091
AST	− 0.401	0.689	1.048	0.295	− 1.664	0.097	− 0.093	0.926

*P < 0.05, **P < 0.01.

## Discussion

The obesity pandemic and the emergence of metabolic syndrome have led to a rise in the prevalence of MAFLD, a global health issue that has a worsening socioeconomic effect^17^. Simple hepatic steatosis can develop into non-alcoholic steatohepatitis, cirrhosis, end-stage liver disease, or hepatocellular carcinoma in the case of MAFLD^18^. An essential part of the pathophysiological mechanism of MAFLD is insulin resistance^19^.

Adipose tissue, an energy storage tissue, is currently regarded as a metabolically active endocrine organ that secretes a range of biologically active chemical molecules known as adipokines, which are crucial for regulating energy metabolism, inflammation, and fibrosis^20,21^. Adipokines are a potential therapeutic target for MAFLD because there is growing evidence that they regulate these metabolic events by modulating insulin-mediated glucose metabolism, fatty acid utilization, and visceral lipid deposition. Adipokine dysfunction is also linked to the development and advancement of MAFLD^22^. Adipocyte fatty acid-binding protein, visfatin, chemerin, retinol-binding protein 4, ghrelin, resistin, leptin, and adiponectin are among the conventional adipokines that have been linked to the emergence of MAFLD in several clinical investigations^23^.

Both the central nervous system and peripheral tissues and organs are affected by asprosin in a variety of ways. Primary target tissues and organs on the periphery include the heart, liver, pancreas, and skeletal muscle, where asprosin can act through proinflammatory responses, oxidative stress, endoplasmic reticulum stress, and apoptosis. The central receptors for asprosin are primarily found in the arcuate nucleus of the hypothalamus, which influences appetite^24,25^. Since its discovery, the focus has been on the association of asprosin with various metabolic diseases. Both clinical and preclinical research have demonstrated that asprosin concentrations are disrupted in individuals with T2DM, polycystic ovarian syndrome, obesity, cardiovascular disease, and several cancers. However, there is a lack of research on the correlation between asprosin and MAFLD.

MAFLD is a crucial element of metabolic disorder, although research is scarce on the correlation between asprosin and MAFLD. According to the results of a study involving children and adolescents, children with MAFLD had elevated levels of asprosin in their blood serum compared to children without MAFLD. In children with MAFLD, ALT, an indicator that quantifies the extent of hepatocellular injury, was also found to be substantially elevated. Thus, the results from the study suggest a strong association between asprosin and the development of MAFLD in children and adolescents^26^. An investigation involving adults revealed that untreated patients diagnosed with MAFLD exhibited notably excessive concentrations of serum asprosin in comparison to individuals in a healthy state. Furthermore, there was a positive association between asprosin levels and various factors such as albumin, FBS, homeostasis model assessment of insulin resistance (HOMA-IR), and TG levels. These were independently correlated with FBS and TG levels. This indicates that asprosin can serve as a non-invasive indicator for predicting the risk of developing MAFLD^27^.

However, the above studies used conventional ultrasound for the qualitative diagnosis of MAFLD, which was highly influenced by the subjective judgment of the examining physicians. In the present study, Fibroscan's elastography technology was used to detect CAP and provide a quantitative assessment of MAFLD, which is more objective and accurate. In addition, the current study had a larger sample size compared to previous studies and included a healthy population undergoing physical examinations, making it more representative of the overall population. It was observed that the levels of asprosin in the serum were substantially elevated in overweight and obese individuals in comparison to the normal weight individuals. Additionally, a significant positive relationship between asprosin and CAP was identified, a marker of hepatic steatosis. Further subgroup analyses revealed that this positive relationship was observed in all three groups, which aligned with the previous research findings. Furthermore, the current research examined the link between serum asprosin levels and different clinical parameters. It was observed that a positive association between asprosin and BMI, WC, TG, TC, LDL-C, FBG, and HbA1c existed. Additionally, asprosin displayed a negative relationship with HDL-C in overweight and obese individuals. The results presented here align with prior research that revealed a positive association between asprosin levels and numerous parameters such as BMI, WC, FPG, 2 h postprandial glucose, HOMA-IR, TG, MCP-1, IL-6, and a negative association with HDL-C in a study group diagnosed with metabolic syndrome^28^. In a population with impaired glucose and lipid metabolism, asprosin levels were positively linked with HbA1c, WC, 2 h postprandial glucose, TG, FBG, and HOMA-IR^29^.

The degree of hepatic steatosis was reflected by the CAP measured by Fibroscan in this study. Most previous studies used conventional abdominal ultrasound to detect hepatic steatosis, which is susceptible to the subjective judgment of the operator and is less sensitive in assessing hepatic fat content below 30%^30^. Fibroscan is a non-invasive and convenient technology that quantitatively assesses steatosis. It has been endorsed by the Asia–Pacific Task Force on Non-Alcoholic Liver Disease recommendations as a screening tool for MAFLD^31^. A study of MAFLD screening in diabetic patients found that high WC, hypertriglyceridemia, and low HDL-L were strongly associated with increased CAP and that the factor with the greatest impact on hepatic steatosis was obesity^32^. Recent investigations have demonstrated a correlation between elevated CAP levels and increased BMI, WC, neck circumference, white blood cell count, waist-to-hip ratio, fasting insulin, uric acid, TG, and HOMA-IR. Additionally, WC and HOMA-IR may be used to predict an elevated risk of developing hepatic steatosis^33^. The findings of this research demonstrated the presence of a positive link of CAP with BMI, WC, asprosin, LSM, TG, TC, LDL-C, FBG, HbA1c, ALT, and AST and a negative correlation with HDL-C. Following a multivariate investigation, it was determined that BMI, WC, asprosin, TG, LDL-C, and HDL-C all independently affect CAP. This confirms the strong association between asprosin and MAFLD.

Considering the correlation between asprosin and metabolic diseases like MAFLD, diabetes, obesity, and polycystic ovary syndrome, asprosin appears to offer novel concepts and perspectives for the clinical diagnosis and treatment of metabolic diseases. Furthermore, it exhibits promise as a new therapeutic target for interventions targeting disorders related to metabolism. The anti-asprosin monoclonal antibody has been acknowledged for its ability to decrease blood glucose levels, suppress appetite, and reduce body weight. It is regarded as a potential treatment strategy for managing diabetes and obesity^34^. However, since the discovery of asprosin in 2016, several unresolved matters remain, such as identifying the precise receptors and processes responsible for the diverse impacts of asprosin on tissues and organs. Therefore, a considerable amount of research is yet needed.

This study has the following advantages. First, this study was conducted in the physical examination population rather than the disease population, which is more reflective of the underlying population; secondly, Fibroscan, a non-invasive and convenient technique, was used to quantitatively assess hepatic steatosis, making it suitable for large-scale screening studies in the population at risk; third, asprosin and MAFLD in the Chinese population were examined for the first time in this study where BMI-based subgroup analyses were carried out. Undoubtedly, this study has certain drawbacks. This study was observational, meaning it cannot establish a cause-and-effect link between asprosin and MAFLD. Additionally, it cannot eliminate the impact of other factors that might affect the results, such as exercise and dietary patterns.

## Data Availability

The data that support the findings of this study are available on request from the corresponding author.
